# Modified technique for trocar-based sutureless scleral fixation of intraocular lenses: A new approach to haptic externalization

**DOI:** 10.1016/j.ajoc.2021.101145

**Published:** 2021-06-16

**Authors:** Gregory J. Bever, Yingna Liu, Jay M. Stewart

**Affiliations:** a University of California, San Francisco, Department of Ophthalmology, San Francisco, CA, USA; b Zuckerberg San Francisco General Hospital and Trauma Center, Department of Ophthalmology, San Francisco, CA, USA

**Keywords:** Haptic externalization, Scleral fixated intraocular lens, Trocar-based sutureless intraocular lens fixation, Secondary intraocular lens

## Abstract

**Purpose:**

To describe a modified technique of haptic externalization for trocar-based sutureless scleral fixation of intraocular lenses, in order to avoid working with forceps in the iris plane in a manner that may be unfamiliar to the vitreoretinal surgeon.

**Methods:**

This prospective, interventional case series included four eyes of four patients with dislocated intraocular lens (IOL). The modified haptic externalization technique avoids the pitfalls of iris-plane maneuvers by intentionally dropping the IOL onto the retina and using the forceps to grasp the tips of the haptics prior to direct externalization from the same grab.

**Results:**

Four patients underwent scleral fixation of IOL using modified haptic externalization technique. One patient was lost to follow up after postoperative day 1. At the last follow up, all eyes demonstrated stability and good centration of scleral fixated IOL. All three patients achieved a best corrected visual acuity same or better compared to before the operation. One patient developed vitreous hemorrhage which later spontaneously resolved.

**Conclusions:**

The modified haptic externalization technique is a simple and quick modification using maneuvers familiar to vitreoretinal surgeons. It has demonstrated safety among a small pilot group of patients.

## 1 Introduction

Since its description by Gabor and Pavlidis,^1^ sutureless scleral-fixated posterior chamber intraocular lens (IOL) techniques have undergone numerous modifications, with improvement in intraoperative safety and efficiency. Using instruments familiar to vitreoretinal surgeons, Prasad^2^ described a technique of creating scleral tunnels using the vitrectomy trocar-cannula system. This technique was later popularized in the United States by Abbey and Williams, whose results demonstrated similar outcomes between the trocar-based technique and the traditional scleral tunnel approach.^3^ Later, Yamane described a flanged haptic technique that prevents the haptic from sliding back into the vitreous cavity causing dislocation. This advancement further improved the stability of scleral-fixated IOLs.^4^

One of the most challenging and time-consuming steps in the learning curve of sutureless scleral fixation of IOLs is haptic externalization. Many of the described methods involve grabbing the haptic tip at or near the iris plane prior to externalization. Working with forceps in this plane may be unfamiliar to the vitreoretinal surgeon and often leads to distortion of the cornea and/or gaping of any clear cornea or scleral tunnel wound. This can cause compromised visualization and even iris prolapse. In addition, methods such as the “handshake” maneuver (whereby a haptic is passed from forceps in one hand to forceps in the contralateral hand prior to externalization) require two forceps, which add cost to the operation. Herein, we introduce a modification of the trocar-based sutureless scleral fixation technique in which haptic work at the iris plane is replaced with maneuvers more familiar to vitreoretinal surgeons.

## 2 Materials and methods

**Fig. 1:**
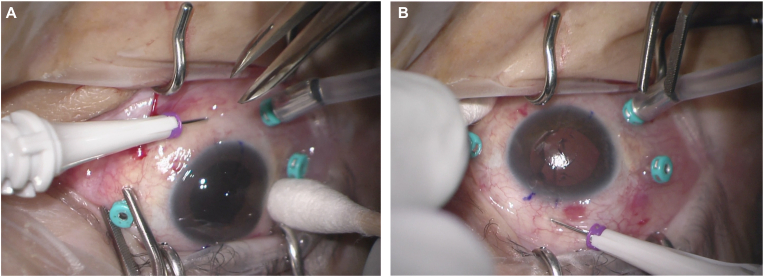
**Scleral tunnel created by trocar-cannula system.** A 27-gauge trocar-cannula is inserted in a clockwise direction, at an acute angle approximately 30° to the ocular surface parallel to and 2.5–3 mm posterior to the limbus, at 6 o'clock (A), and 12 o'clock (B).

Placement of a three-port 25-gauge trocar-cannula system is first conducted in the usual manner for vitreoretinal surgeries. The infusion line cannula is placed inferotemporally, and the two cannulas for working instruments are placed superotemporally and superonasally. All three of these cannulas are moved closer to the horizontal meridian than is typically done to allow sufficient space for the two haptic externalization cannulas at 6 and 12 o'clock. A complete pars plana vitrectomy is then performed with careful attention paid to vitreous base shaving. If the vitreous base is not sufficiently shaved, the haptics may get caught in vitreous during the later externalization steps. Removing any residual lens capsule at this time is also helpful to prevent haptic incarceration in capsule. A toric marker is then used to mark the limbus at 6 and 12 o'clock. Aiming for an acute angle approximately 30° to the ocular surface, a 27-gauge trocar-cannula is inserted in a clockwise direction at 6 o'clock parallel to and 2.5–3 mm posterior to the limbus (Fig. 1A). This creates a scleral tunnel 2–3 mm in length inferiorly. The trocar is removed, leaving the cannula in place. A second trocar-cannula system is placed in the same manner, creating a second scleral tunnel exactly 180° away, at the previously marked 12 o'clock location (Fig. 1B). Clockwise entry of both the 6 and 12 o'clock haptic externalization cannulas allows for proper IOL orientation.

**Fig. 2:**
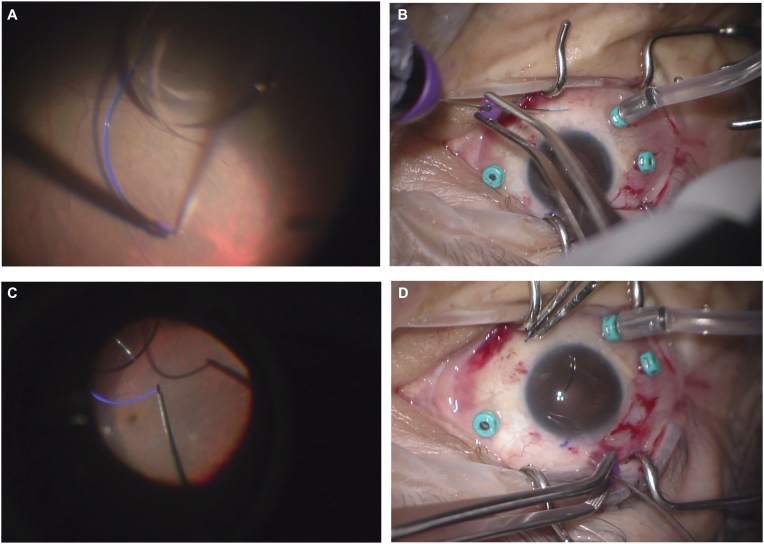
**Leading haptic externalization.** After the intraocular lens was intentionally dropped onto the retina in a “Z" configuration, a pair of 27-gauge serrated forceps is used to grasp the tip of IOL haptic (A). The haptic is then externalized directly from this grab. But before pulling the haptic out of the eye, the cannula is gently pulled back over the forceps shaft (B), allowing the haptic to seat in the scleral tunnel. The same 27-gauge serrated forceps is then inserted through the other cannula and used to grasp the tip of the second haptic now suspending in the vitreous cavity (C). This trailing haptic is then externalized directly through the grab, while gentling pulling back the cannula over the forceps shaft (D).

Next, a scleral tunnel is created superotemporally to allow insertion of a three-piece intraocular lens (IOL). The authors prefer a scleral tunnel wound, though a clear corneal incision could be considered if it is necessary to minimize scleral and conjunctival involvement. When the IOL is inserted, it is intentionally dropped onto the posterior pole of the retina. It is important for the IOL to be oriented in the correct “Z” orientation at this step. If in the “S” orientation, it should be flipped. Using a non-contact viewing system or other standard method for intraoperative visualization of vitreoretinal surgery, a pair of 27-gauge serrated forceps is inserted through the 6 o'clock 27-gauge cannula and used to grasp the tip of either IOL haptic (Fig. 2A). The haptic is then externalized directly from this grab without any work in the iris plane. Before pulling the haptic out of the eye, the cannula is gently pulled back over the forceps shaft (Fig. 2B). This seats the haptic in a tight scleral tunnel as it is externalized. Thermal cautery is applied to the tip of the externalized haptic, creating a flange for securement. The haptic can then be pushed back into the scleral tunnel so that the flange sits flush with the sclera. The non-contact viewing system is then used to visualize the trailing second haptic, which is now hanging in the middle of the vitreous cavity and well-presented for the second forceps grab. The same 27-gauge serrated forceps is then inserted through the 12 o'clock 27-gauge cannula and used to grasp the tip of the second haptic (Fig. 2C). This trailing haptic is then externalized and cauterized in the same manner (Fig. 2D). If needed, additional cautery can be applied to one or both haptics to optimize IOL centration. A complete demonstration of the modified technique for lens implantation and fixation can be seen in the attached video.

## 3 Results

This technique has been performed in four eyes of four patients. The preoperative and postoperative corrected visual acuity is shown in Table 1. Of note, most of these procedures were performed in a tertiary center in which patients had concurrent ocular diseases that limited visual potential and/or psychosocial conditions that prevented reliable follow-up. No intraoperative complications were encountered; specifically, dropping the IOL onto the posterior pole did not result in retinal injury in any patient included in this study.

**Table 1:** Postoperative outcomes after scleral fixation of intraocular lens using modified technique for haptic externalization.

Patient age and sex	Preoperative corrected vision	POD1 vision	POW1 vision	POM1 vision	Most recent vision	Lens stability	Notes/Postoperative complications
58 year old man	HM	HM	HM	HM	HM	Stable, centered	Visual potential limited by foveal outer retinal atrophy. History of fovea-off retinal detachment
58 year old man	20/150	CF				Unknown	Lost to follow up after POD1 visit
64 year old man	20/50	20/200	CF	20/150	20/40	Stable, centered	Patient developed postoperative vitreous hemorrhage noted on POW1 visit that subsequently resolved
48 year old man	LP	Unable	Unable	20/400	20/400	Stable, centered	Unstable mental status, visual acuity very difficult to reliably measure

CF = counting fingers, HM = hand motion, LP = light perception, POD1 = postoperative 1 day, POW1 = postoperative 1 week, POM1 = postoperative 1 month.

Overall, among the three patients who had adequate follow up for at least 1 month after scleral-fixated IOL procedure using the described technique, all demonstrated lens stability during the most recent visit. One patient experienced postoperative vitreous hemorrhage that resulted in vision decline one week postoperatively, which subsequently resolved, and vision recovered back to baseline and better at the most recent visit, 2.5 months after surgery.

## 4 Discussion

Our technique uses visualization strategies and forceps maneuvers that are routine and familiar to vitreoretinal surgeons rather than potentially unwieldy, time-consuming iris-plane maneuvers that are less commonly performed by posterior segment surgeons. In addition, this strategy can be particularly useful in situations in which there is poor pupillary dilation, or when the pupil constricts during surgery, because modern operating microscope systems for vitreoretinal surgery allow sufficient visualization of each haptic tip in the vitreous cavity, one on the retinal surface and the other in the mid-vitreous. Furthermore, IOL fixation can be achieved with minimal instrumentation; specifically, only one pair of forceps is needed, making this approach more cost-effective than “handshake” techniques. Future iterations of this technique could reduce cost even further by eliminating the need for the 27-gauge cannulas, if the forceps are introduced directly into the eye through scleral tunnels without pre-placement of trocar-cannulas. We believe our technique improves efficiency and adds to the previously described methods of sutureless scleral fixation of IOLs.

Our technique has potential limitations. Dropping an IOL onto the posterior pole of the retina can theoretically be associated with retinal injury, but it was not encountered in our case series. Placement of a perfluorocarbon liquid bubble in the posterior pole could protect against possible retinal injury, but the authors did not find this to be necessary. In addition, because this technique involves grasping the tip of the IOL haptic over the retina, it limits the adoption of this technique by non-vitreoretinal trained surgeons.

## 5 Conclusions

The modified haptic externalization technique is a simple and quick modification using maneuvers familiar to vitreoretinal surgeons. It has demonstrated safety among a small pilot group of patients.

## Declaration of competing interest

The authors declare that they have no conflicts of interests.

## Appendix A Supplementary data

A toric marker is used to mark the limbus at 6 and 12 o'clock. Aiming for an acute angle approximately 30° to the ocular surface, two 27-gauge trocar-cannulas are inserted in a clockwise direction at 6 and 12 o'clock parallel to and 3 mm posterior to the limbus. A scleral tunnel is created superotemporally to allow insertion of a three-piece intraocular lens (IOL). The IOL is then intentionally dropped onto the posterior pole of the retina in a “Z” configuration. A single pair of 27-gauge serrated forceps is used to grasp the tip of one of the haptics on the retinal surface and directly externalize it without any work in the iris plane, and then the second haptic is grasped while hanging in the mid-vitreous cavity and externalized in similar fashion.
